# Antibiofilm peptides: overcoming biofilm-related treatment failure

**DOI:** 10.1039/d0ra09739j

**Published:** 2021-01-13

**Authors:** Melanie Dostert, Michael J. Trimble, Robert E. W. Hancock

**Affiliations:** Department of Microbiology and Immunology, University of British Columbia Vancouver British Columbia Canada bob@hancocklab.com

## Abstract

Health leaders and scientists worldwide consider antibiotic resistance among the world's most dangerous pathogens as one of the biggest threats to global health. Antibiotic resistance has largely been attributed to genetic changes, but the role and recalcitrance of biofilms, largely due to growth state dependent adaptive resistance, is becoming increasingly appreciated. Biofilms are mono- and multi-species microbial communities embedded in an extracellular, protective matrix. In this growth state, bacteria are transcriptionally primed to survive extracellular stresses. Adaptations, affecting metabolism, regulation, surface charge, immune recognition and clearance, allow bacteria to thrive in the human body and withstand antibiotics and the host immune system. Biofilms resist clearance by multiple antibiotics and have a major role in chronic infections, causing more than 65% of all infections. No specific antibiofilm agents have been developed. Thus, there is a pressing need for alternatives to traditional antibiotics that directly inhibit and/or eradicate biofilms. Host defence peptides (HDPs) are small cationic peptides that are part of the innate immune system to both directly kill microbes but also function to modulate the immune response. Specific HDPs and their derivatives demonstrate broad-spectrum activity against biofilms. *In vivo* biofilm assays show efficacy in abscess, respiratory, in-dwelling device, contact lens and skin infection models. Further progress has been made through the study of *ex vivo* organoid and air–liquid interface models to better understand human infections and treatment while relieving the burden and complex nature of animal models. These avenues pave the way for a better understanding and treatment of the underlying cause of chronic infections that challenge the healthcare system.

## Abstract 2

Host defence peptides (HDPs) can overcome biofilm-specific resistance and immune evasion mechanisms that render antibiotics ineffective. Infection models mimicking the resilience of biofilms are crucial for the development of these therapeutics.

## Graphical abstract

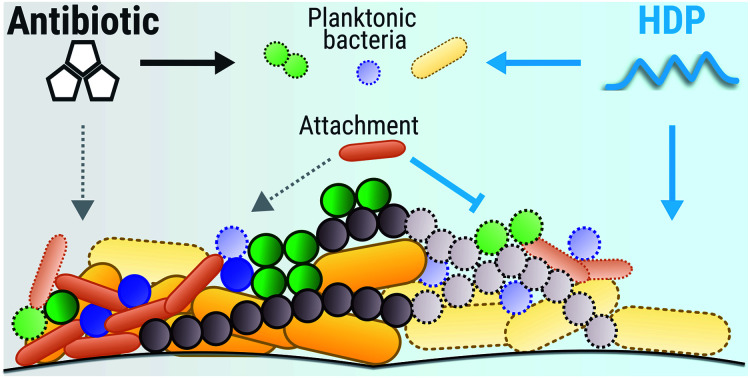

## Highly antibiotic resistant biofilm infections

Antimicrobial resistance describes the failure of antibiotics to clear infections due to the ability of microorganisms to survive exposure to drugs that were developed to kill them.^1^ First reports of antimicrobial resistance arose more or less simultaneously with the clinical application of antibiotics. Subsequently, decades of antibiotic overuse in healthcare and agriculture have led to antibiotic accumulation in the environment, which ultimately created a vicious cycle and a gradually exacerbating crisis.^1^ Today, our hopes are on last resort antibiotics, and conservative estimates have already attributed 700 000 worldwide deaths per year to resistant or multidrug resistant (MDR) bacteria.^2^ However, the actual situation is already far worse. In 2017 more than 11 million people died from sepsis,^3^ which is a dysregulated host response to infection and represents a classic case of antibiotic failure in form of effective therapeutic resistance, since antibiotics are the major front line treatment for sepsis.

Indeed, antimicrobial resistance is a complex and mechanistically diverse process. In clinical settings, antibiotic therapy frequently fails to cure infections caused by microbial communities termed biofilms, which represent the majority (>65%) of all infections in humans.^4,5^ Biofilm infections are largely chronic, typically remain local, show less pronounced but prolonged inflammatory symptoms, evade immune clearance, resist antibiotics and are often poorly accessible for sampling.^6–8^ Combined with a lack of reliable, standardized methods for clinical detection, physicians not only struggle to treat but also to diagnose biofilm infections.^8^ Biofilms are communities of microorganisms, usually adherent to surfaces, and embedded in an extracellular matrix.^4,6^ They are often considered to represent adaptations to stress but in effect they are likely the most common growth mode for microorganisms in the environment and can occur throughout the body. Clinically relevant biofilms manifest in various forms ranging from surface-associated communities on tissues or medical devices, which are by far the most common, to aggregates within mucus layers and even intracellular clusters.^4^ These phenotypic variants appear to be a consequence of the microenvironment at the site of infection, including exposure to antimicrobial and host defence stresses. Biofilms can comprise a single organism or complex polymicrobial communities and either contribute to overall human health or cause infections.^7,9^ Although traditionally it was suggested that only pathogens cause infections, recent studies increasingly associate disease progression with the loss of microbial diversity in health-promoting polymicrobial communities.^7,9^

In diverse environments, including surfaces in the human body, microorganisms undergo extensive adaptive (environment-dependent but reversible) changes in gene expression that are controlled by dynamic and complex interconnected regulatory networks.^4^ The biofilm life cycle follows a developmental program of multiple sequential stages with the current model being substantially based on *in vitro* observations of surface-attached *Pseudomonas aeruginosa* biofilms.^10^ Triggered by environmental cues, planktonic bacteria first adhere reversibly to a surface before producing extracellular matrix components. These then irreversibly attached microcolonies lay the foundation for the following maturation steps, in which the biofilm community grows through cell division and develops a three-dimensional architecture. Lastly, individual cells or small clusters can disperse from the mature biofilm and reinitiate the life cycle. This is a very important stage of the life cycle since it allows localized biofilm infections (*e.g.* on catheters or body surfaces/wounds) to lead to systemic infections. Microorganisms growing in biofilms exhibit both adaptive resistance to almost all antimicrobials by 10 to 1000 fold and increased resistance to the host immune system when compared to their free-living, planktonic counterparts.^4,11^ Moreover, sub-inhibitory concentrations of antibiotics have the ability to stimulate and enhance biofilm establishment, creating a potentially vicious cycle.^12^ Furthermore, it is broadly accepted that the spatial proximity of microorganisms in biofilms facilitates other resistance mechanisms such as horizontal gene transfer and genetic diversification.^5^

Despite the significant health and economic burden of biofilm infections, there are absolutely no selective antibiofilm drugs available, which leaves clinicians with very few therapeutic options. Clinical treatment is often rather aggressive and typically includes surgical removal of colonized medical devices or tissues, termed debridement, as well as long-term therapy with high doses of multiple antibiotics combined.^4^ To overcome frequent therapeutic failure and infection recurrence, innovative treatment strategies need to target biofilm-specific adaptive resistance mechanisms.

## Biofilm-specific resilience mechanisms

Therapeutic clearance of microbial infections is the result of an effective interplay between antibiotics and host responses.^13^ Whereas infections caused by planktonic bacteria are typically sensitive to antibiotics and host defences, the combination of antimicrobial adaptive resistance and various immune evasion mechanisms, which we collectively term resilience, renders treatment of biofilm infections difficult and often ineffective.^11,13^ The underlying mechanisms are complex, interconnected and inadequately understood. Here, we emphasize the role of biofilm-specific gene expression, regulatory and metabolic heterogeneity, extracellular matrix, as well as polymicrobial and host–biofilm interactions.

### Gene expression reprogramming drives resistance in biofilms

Microorganisms growing in biofilms exhibit distinct gene expression patterns when compared to their planktonic counterparts, which we have previously described as a biofilm “program”, determining the physiology, responses and adaptive abilities (lifestyle) of biofilms.^14^ Thus, to explain their protective nature, it is critical to identify genes preferentially expressed in biofilms.^11,15^ These lifestyle-specific changes in gene expression involve substantial proportions of all genes, are likely specific to bacterial species, environment and developmental stage/age, and encompass genetic determinants for biofilm growth and physiology as well as inherent biofilm properties, such as stress management, adaptive antimicrobial resistance and immune evasion. To date, there are relatively few reports of genes underlying biofilm-specific resistance or immune evasion mechanisms, making this an active area for future studies.

Biofilm-specific adaptive resistance has largely been studied in *P. aeruginosa*. Nucleotide signaling in the form of the stringent stress response is a major regulatory pathway driving adaptive biofilm resistance in several ways. The stringent stress response, identified in many bacteria as required for biofilm formation, under at least some conditions,^16^ relies on the alarmone guanosine tetraphosphate (ppGpp), and its precursor guanosine pentaphosphate, which act on the RNA polymerase to regulate the expression of numerous genes.^11^ The two enzymes RelA, responsive primarily to amino acid starvation, and SpoT, that responds to many other stresses, regulate the intracellular levels of ppGpp in *P. aeruginosa* and other Gram-negative bacteria.^15^ Studies suggest that both enzymes are required for biofilm resistance to multiple antibiotics with different cellular targets, acting through impaired antioxidant defences and increased pro-oxidant production.^11,17,18^ In addition, ppGpp signaling has been linked to the formation of highly recalcitrant persister cells.^13,19^ Persisters are phenotypic variants that are dormant in the presence of antibiotics but can resume growth exhibiting non-persister susceptibility profiles in the abscence of antibiotic pressure.^11,15^ Even though persisters represent only a minor subpopulation of the biofilm community (<1%), they are believed to be important for infection recurrence after antibiotic treatment in the clinics.^13^

One of the best understood mechanisms of resistance is mediated by extracellular DNA (eDNA) in the biofilm matrix.^20^ It has been suggested that eDNA creates acidic microenvironments and also decreases the effective concentrations of cations by chelation.^20,21^ These environmental signals trigger an adaptive process that ultimately masks the negative charge of the bacterial surface and consequently reduces self-promoted uptake, leading to resistance to polycationic antimicrobials, such as polymyxins, aminoglycosides and antimicrobial peptides.^20^ The presence of negatively charged eDNA activates the two-component regulatory systems PhoPQ and/or PmrAB,^20,21^ which regulate the expression of the *arn* and spermidine biosynthesis clusters, leading to lipopolysaccharide (LPS) modification and the production of exogenous as well as membrane-associated polyamines, respectively.^20^

Screening of a transposon mutant library for genes influencing antimicrobial susceptibility against tobramycin in biofilms, but not planktonic growth, identified among others the *ndvB* gene, which is preferentially expressed in biofilms and encodes a glucosyltransferase required for the biosynthesis of periplasmic glucans.^15^ Based on the observation that tobramycin only interacted with ethanol-extracted periplasmic material from wildtype but not from a *ndvB* deletion mutant, it was proposed that periplasmic glucans sequester antibiotics and either prevent or slow antibiotic diffusion into the cytoplasm of biofilm bacteria. Comparable to the barrier function of the biofilm matrix, sequestration of antibiotics in the periplasm might allow bacteria to activate protective mechanisms and survive antibiotic exposure. In a follow-up study, *ndvB*-derived periplasmic glucans were also linked to biofilm-specific adaptive resistance through activation of ethanol oxidation genes in the absence of inducers, like ethanol or other alcohols.^15^ Other adaptive resistance genes preferentially expressed in *P. aeruginosa* biofilms include an ATP-binding cassette (ABC) transporter PA1875–77, the type VI secretion system gene *tssC1*, two-component regulator PA0756–57 and two hypothetical genes PA2070 and PA5033.^15^

Furthermore, the expression of *ndvB* and the ABC transporter PA1874–77 was linked to the MerR-like transcriptional regulator BrlR, that plays a role in *P. aeruginosa* adaptive resistance to multiple classes of antibiotics (but not biofilm development *per se*).^15,22^ Global transcriptomic analysis showed that biofilm-specific expression of *brlR* not only activated these previously identified adaptive biofilm resistance genes but also the efflux pumps *mexAB-oprM* and *mexEF-oprN* as well as seven additional ABC transporters.^15,22^ The activity of BrlR itself, as confirmed by pull down and cross-linking experiments, depends on its dimerization state, which is modulated by the intracellular second messenger cyclic diguanylate (c-di-GMP),^23^ a key regulator of the biofilm life style, as detailed below. In this vein, expression levels and DNA-binding capability of the transcriptional regulator BrlR were specifically linked to the c-di-GMP synthetase PA3177, which itself does not impact biofilm formation only adaptive resistance.^24^ Moreover, crystal structures of BrlR revealed that this transcriptional regulator is not only modulated by c-di-GMP but also by the virulence factor pyocyanin, another important regulator of biofilm growth.^25^

Recently, biofilm-specific adaptive resistance in group A *Streptococcus* was linked to the arginine deiminase pathway encoded by the *arc* operon.^26^ Inactivation of this operon specifically increased *in vitro* biofilm susceptibility to multiple classes of antibiotics without affecting biofilm growth. Planktonic levels of antibiotic susceptibility observed in *arc* mutant biofilms were associated with an inability to respond to acid stress. Correspondingly, the loss of the *arc* operon significantly improved the treatment effect of intramuscularly injected penicillin G in a murine nasopharyngeal infection model. Interestingly, *arc* mutant biofilms formed smaller clusters when compared to the dense wild type microcolonies, without affecting overall bacterial density *in vivo*.

Beyond adaptive resistance genes preferentially expressed in the biofilm growth state, some reports have also identified genes that confer biofilm-specific protection without displaying differential expression between biofilm and planktonic growth. Examples include the glycosyltransferases EpaI and EpaOX in *Enterococcus faecalis* that are involved in the biosynthesis of cell wall-associated polysaccharides and biofilm architecture^27,28^ and β-lactamases that are more concentrated and work better in the discrete microenvironment of *Pseudomonas* biofilms.^29^ Furthermore, there are many genes that are involved in the stimulation of biofilm formation in the presence of subinhibitory concentrations of antibiotics.^30^

To our knowledge, reports of genes that are preferentially expressed in biofilms and promote immune virulence, are uncommon. Indeed, the chronic biofilm state is usually considered to be less virulent (albeit inducing sustained inflammation). Biofilms are usually considered to physically limit the activities of phagocytic cells, as outlined below in the discussion of the biofilm matrix. However, two independent studies, reported that *Staphylococcus aureus* biofilm supernatants are significantly more effective at killing phagocytes than media collected from planktonic cultures.^31,32^ Using proteomics, α-toxin and bicomponent leukotoxin AB (LukAB) were identified as key synergistic factors in mediating biofilm-selective cytotoxicity against macrophages *in vitro* and in a murine orthopedic implant biofilm infection model.^32^ It was demonstrated that only simultaneous deletion of both Panton–Valentine leukocidin and α-hemolysin significantly reduced biofilm-mediated neutrophil-killing, by inducing NET formation without affecting biofilm survival *in vitro*, and reduced infection in a porcine model of chronic burn wound infection.^31^

### Regulatory and metabolic adaptations promoting biofilm heterogeneity

Biofilm growth creates a heterogeneous microenvironment, in which microorganisms are exposed to distinct niches and selective pressures depending on their positioning within the biofilm.^11,15,33^ Increasing biomass and three-dimensional complexity during biofilm maturation leads to the formation of steeply decreasing nutrient and oxygen gradients from the periphery to the center.^11,15,33^ Various physical and chemical conditions within the microbial community lead to heterogeneous activation of regulatory and metabolic systems, which promotes the emergence of highly resilient subpopulations.^33^ Physiological adaptations, genomic mutations and horizontal gene transfer can give rise to these diverse subpopulations with enhanced resistance.^13,19^ Below we discuss the role of transient regulatory and metabolic adaptations in biofilm resilience.

In biofilms, microorganisms dynamically change gene expression and a variety of signaling systems has been implicated in biofilm regulation on transcriptional, post-transcriptional and post-translational level.^34^ Many of these regulatory systems not only initiate, maintain or disperse biofilm growth, but also actively drive the associated resistance phenotypes. Several two-component regulators that mediate biofilm resistance mechanisms were discussed above, and such regulators that drive rapid adaptation to environmental changes (such as nutrient availability) likely play an important role in regional and whole-biofilm adaptive resistance, in addition to related phenomena such as tolerance and persistence. Other important regulatory mechanisms include second messenger nucleotide-based signaling systems, stress responses and metabolism.

An important nucleotide signaling pathway involved in the biofilm lifestyle involves the widely-distributed second messenger c-di-GMP, which promotes biofilm formation at high intracellular levels and favours planktonic growth at low intracellular levels.^35^ In *P. aeruginosa* numerous diguanylate cyclases and phosphodiesterases control the intracellular levels of c-di-GMP.^35^ Through interaction with its cognate receptors, c-di-GMP can mediate riboswitch regulation, activate transcription factors, such as the previously mentioned BrlR in *P. aeruginosa*, and modulate enzyme activity.^35^ Using a bacterial two-hybrid screen, WarA was identified as an interactor of the diguanylate cyclase SadC, which promotes irreversible attachment in *P. aeruginosa*.^36^ The methyltransferase WarA was shown to specifically bind c-di-GMP, potentiating its activity.^36^ Deletion of *warA* increases both average LPS chain length *in vitro* and survival in a zebrafish infection model due to enhanced neutrophil recruitment and higher transcript levels of the pro-inflammatory cytokine tumor necrosis factor alpha.^36^ Therefore, the authors hypothesized that SadC and WarA protect *P. aeruginosa* from host responses by regulating LPS chain length.^36^ However, the relevance of this c-di-GMP-mediated immune evasion mechanism remains to be proven during the biofilm growth state itself. Conversely, increasing the levels of c-di-GMP in planktonic cells to those levels experienced in biofilms, led to significantly higher resistance to various antimicrobial agents, with this resistance likely being mediated by SagS.^37^ Beyond their established intracellular function, nucleotide second messengers are also able to adopt extracellular roles to promote biofilm immune evasion, as demonstrated for cyclic diadenylate (c-di-AMP).^38^ This cyclic dinucleotide is predominantly produced in Gram-positive bacteria and its intracellular levels are controlled by relatively few enzymes.^39^ It was demonstrated that *S. aureus* biofilms release up to 60% of total c-di-AMP by autolysis, which in return dose-dependently induces the expression of interferon-β and interleukin-6 in macrophages.^38^ This immune evasion mechanism is biofilm-specific and triggers a type I interferon response through c-di-AMP binding to the stimulator of interferon genes.^38^ The authors suggested that c-di-AMP release by extracellular *S. aureus* biofilms might promote persistence by skewing macrophages towards an anti-inflammatory state,^38^ but this appears to be inconsistent with the observation that biofilms often trigger chronic inflammatory responses. Further supporting an extracellular role, Li *et al.* showed that c-di-GMP binding inhibits the bacteriostatic activity of recombinant human siderocalin, an innate immunity protein that sequesters bacterial iron acquiring siderophores.^40^

The universal stringent stress response based on the second messenger nucleotide ppGpp, as outlined above, mediates biofilm formation and antibiotic resistance/persistence, but more specifically since this regulatory mechanism responds to nutrient and oxygen deprivation as occurring in the deeper layers of biofilms, it likely has important effects on regional susceptibility and persistence. Similarly to the stringent stress response in *P. aeruginosa*, activation of oxidative stress response genes mediated by the transcriptional regulator SpxA1 has also been implicated in biofilm adaptive resistance in *Streptococcus mutans*.^41^

Transcriptomic approaches comparing biofilm and planktonic growth of *P. aeruginosa* have revealed activation of regulons under the control of master regulators like RpoS, Anr, VqsM and MvfR.^17,18^ The enhanced susceptibility of mutants lacking either the stationary phase sigma factor RpoS, the global anaerobic regulator Anr, or the membrane stress regulator AmgR indicates that metabolic adaptations in response to hypoxia and stress contribute to adaptive resistance of biofilms to ciprofloxacin.^17,18^ In particular, hypoxia occurring deep within the biofilm, has been proposed to promote adaptive biofilm resistance in several ways. On the one hand, the paucity of oxygen reduces endogenous oxidative stress and might induce expression of efflux pump genes, as demonstrated for MexEF-OprN in planktonic cultures.^15,19^ On the other hand, the low metabolic rates observed during hypoxia slow down growth which reduces susceptibility to antibiotics that depend on cellular growth and energization, including most β-lactams, aminoglycosides and fluoroquinolones.^15,33^ In order to sustain metabolic activity in the hypoxic regions of biofilms, *P. aeruginosa* can use nitrate or phenazines as alternate electron acceptors by upregulating denitrification and phenazine biosynthesis pathways.^15,18,42^ Phenazine reduction mediated by the *cbb3*-type terminal oxidases Cco1 and Cco2 leads to the formation of a distinct metabolically active subpopulation in the hypoxic region of the biofilm;^42^ intriguingly, protecting biofilms against ciprofloxacin.

### The extracellular matrix as a protective barrier

Microbes in biofilms are embedded in a self-produced matrix, which acts as both scaffold and protective barrier.^43^ The extracellular matrix typically contains exopolysaccharides as well as varying levels of extracellular nucleic acids, lipids and proteins, and its exact molecular composition and architecture is highly dependent on the microbial species as well as incorporated host factors and nutrients at the site of infection.^11,43,44^ Representing up to 90% of the volume of mature biofilms, the extracellular matrix substantially increases the total biomass of microbial communities.^12^ Indeed, the way biofilms are structured impacts the efficiency of host responses by triggering a phenomenon termed frustrated phagocytosis.^45,46^ Phagocytes, such as neutrophils and macrophages, are recruited to the site of infection as part of the innate immune response. Receptor-mediated recognition of microbial surface structures activates phagocytes, which then engulf and digest microbes. However, the obscuring of individual cells by the matrix of biofilms leads to steric hindrance of their engulfment by phagocytes, thus impeding microbial clearance.^45,46^

Specific biofilm matrix components can also affect antimicrobial responses of neutrophils, including reactive oxygen species (ROS) and neutrophil extracellular trap (NET) formation. For example, expression, in *S. typhimurium*, of the Vi antigen matrix component of *Salmonella typhi* leads to decreased ROS production in a neutrophil cell line.^47^ Furthermore, *P. aeruginosa* biofilms contain the matrix-associated protease inhibitor ecotin that inhibits neutrophil elastase, a granule protease secreted by neutrophils and often associated with NETs.^48^ Enhanced by its interaction with the exopolysaccharide Psl ecotin is accumulated in the matrix over time, even though it is expressed in both planktonic and biofilm bacteria.^48^ Other membrane-associated structures, such as polymeric-*N*-acetylglucosamine in *Staphylococcus epidermidis*, can also be secreted into the biofilm matrix to act as decoy for immune cell activation.^46,49^

The high viscosity, net charge and abundance of diverse matrix components can limit the diffusion of humoral factors and antibiotics.^12,46^ Charged matrix components, such as eDNA, can sequester antimicrobials through electrostatic interactions and prevent them from reaching microbicidal concentrations.^12^ Consequently, addition of exogenous DNA has been shown to protect *P. aeruginosa* biofilms against the positively-charged antibiotic tobramycin.^50^ However, the observed resistance phenotype cannot be entirely explained by the physical barrier effects of the biofilm matrix and the other factors discussed above are likely equally or even more important. Multiple studies have shown that the impact of the biofilm matrix on diffusion is antibiotic- and strain-specific.^12,13^ Rather than preventing antibiotic penetration altogether, the biofilm matrix likely attenuates diffusion, which exposes microorganisms in deeper biofilm layers to antibiotic concentrations that increase only gradually, thus giving them more time to adapt.^12^

### Host–biofilm and polymicrobial interactions driving biofilm resilience

Most *in vitro* studies focus on single-species, surface-attached biofilms with large three-dimensional structures, even though biofilms in the context of chronic human infections can be more complex. They often involve small aggregates comprised of multiple different species, associated with host cell substrates or embedded in host-derived components (*e.g.* the mucous blanket of epithelial layers), and surrounded by immune cells and other host factors.^33,45,51^ In addition to individual bacterial factors and nutrient availability, biofilm resilience is shaped by immune factors as well as cooperative and competitive polymicrobial interactions.^51^ Conversely, biofilms themselves skew immune cell responses to promote persistence.

As part of the inflammatory response, innate immune cells, particularly neutrophils, are recruited to the site of infection and their active oxygen consumption contributes to a hypoxic microenvironment that can serve as an environmental signal to dramatically enhance biofilm growth.^52^ In the presence of innate immune cells, *P. aeruginosa* biofilms induce production of extracellular rhamnolipids, which can quickly lyse immune cells leading to a chronic inflammatory environment and enhanced biofilm growth due to the release and/or incorporation of intracellular, eukaryotic factors, like proteases, DNA and actin.^45^

Biofilms can skew immune responses through secreting metabolites, triggering gene expression in epithelial cells, reprogramming immune cells, such as macrophages and T cells, as well as recruiting myeloid-derived suppressor cells (MDSCs).^45,53,54^ For example, lactate biosynthesis by *S. aureus* biofilms promotes persistence through inhibition of histone deacetylase-11 leading to enhanced production of the anti-inflammatory cytokine interleukin-10 (IL-10) in MDSCs isolated from a murine prosthetic joint infection model.^55^ Detection of elevated levels of d-lactate and IL-10 in the synovial fluid of patients with prosthetic joint infection supports the relevance of this mechanism in human biofilm infections.^55^ Furthermore, *S. aureus* biofilm supernatants have been shown to impair bactericidal and pro-inflammatory responses in murine macrophage-like cells.^56^ Increased expression of Kruppel-like factor 2 in response to an unknown, secreted bacterial factor leads to reduced activation of the transcription factor NF-κB, which controls expression of multiple immune response genes, including the pro-inflammatory cytokines IL-6 and IL-1β.^56^

In the last decade, increasing studies have focused on multispecies interactions within polymicrobial communities and their role in biofilm resilience.^57^ Secreted factors, such as matrix components produced by other microbial species in the same community, are broadly accepted to provide cross-protection against antibiotics.^57^ In recent years, biofilm resilience mechanisms driven by metabolic interactions between different species have been increasingly investigated.^57^ For example, *Aggregatibacter actinomycetemcomitans* senses hydrogen peroxide production by *Streptococcus gordonii*, which leads to the expression of the complement resistance protein ApiA, which provides protection against serum-mediated killing.^58^ Using a global transcriptomic approach, Yadav *et al.* compared the effect of single and multispecies biofilms in an otitis media rat model.^59^ 222 unique genes differentially expressed in the mucosa simultaneously colonized with *P. aeruginosa* and *S. aureus* included genes involved in immune responses, inflammation, signaling and development, suggesting multispecies communities induce a unique host response compared to single-species biofilms.^59^

## Defining the requirements for successful biofilm therapies

To develop successful therapies, the desirable properties and biological functions of an ideal antibiofilm drug must be first defined. To date, therapies have been adapted from agents (*e.g.* antibiotics) that kill planktonic bacteria and, given the substantial adaptive multi-drug resistance of biofilms, often combinations of agents are used with mixed success. However, given that the physiology of biofilms is substantially different from that of planktonic cells, it makes sense that there should be compounds that preferentially act against one or more stages of the biofilm life cycle.^14^ However, there are additional characteristics that should be taken into account that may vary from those relevant to systemic infections by bacteria growing planktonically as individual units. These include accessibility of biofilms. One important feature of biofilms is that they are often localized in a particular area of the body and are potentially accessible to topical treatment enabling such measures as direct application of the drug onto the biofilm using *e.g.* creams, foams, aerosols, gels, lotions or ointments. A second strategy that is already employed, given the difficulty of treatment of deep-seated, high density biofilm infections is surgical removal/debridement of most of the biofilm followed by treatment of the remaining bacteria to prevent biofilm regrowth in diseases like chronic sinusitis.^60^

There are other factors that must be taken into account. Due to the potential presence of multiple microbial species in biofilm infections, an ideal antibiofilm drug should have broad-spectrum activity against clinically relevant bacteria.^61^ As discussed above, bacteria in biofilms exhibit a variety of metabolic and regulatory programs. Thus, it seems reasonable that antibiofilm agents should be able to target various biofilm-specific structures and mechanisms to ensure efficacy against established, heterogeneous biofilms. In this context, efficacy against persister cells is of particular importance to prevent infection recurrence. Targeting microorganisms in both the biofilm and planktonic growth states seems to be a promising strategy to treat biofilm infections and prevent dissemination into other tissues and the establishment of systemic infection. This can be achieved with a combination of drugs directed against biofilms, for which no specific drugs are currently available, and planktonic cells, prospectively using our existing array of antibiotics. Lastly, the high selectivity of antibiofilm drugs against microorganisms without affecting human cells is essential for therapeutics directed against bacterial infections. However, immunomodulatory activities redirecting misguided immune responses that cause chronic inflammation and can harm host tissues, thus promoting biofilm growth and persistence, would likely improve the outcome and efficacy of antibiofilm drugs.

Rather than providing a comprehensive overview of the various antibiofilm strategies currently under investigation, we would like to refer the reader to a prior review on this topic^62^ and instead highlight the potential of antibiofilm peptides as therapeutics for biofilm infections.

### Antibiofilm peptides

Antibiofilm peptides are derivatives of host defence (also termed antimicrobial) peptides (HDPs), which are essential components of innate immunity in all complex species of life.^63–65^ They are now recognized to have important activities in host defence, including activities in the modulation of the immune response (*e.g.* promotion of protective immunity and anti-inflammatory activity), direct killing of planktonic bacteria, fungi, and parasites, and broad-spectrum antibiofilm activity. These topics have been previously reviewed by us and others,^63,65–67^, so here we provide a summary. The various activities of host defence peptides are independently determined since the structure/activity relationships for these different biological functions overlap but are by no means identical.^68,69^ Indeed, it was demonstrated very early on that a human HDP LL-37, with potent immunomodulatory activity, was able to act against biofilms at concentrations less than one sixteenth of those required to kill planktonic bacteria.^4^ Furthermore, synthetic mimetic peptides that had no activity against planktonic *Burkholderia* sp., were able to inhibit biofilms at low concentrations.^16^ It was subsequently demonstrated that a subset of cationic HDPs and synthetic mimetics can act against biofilms formed by multiple species of bacteria.^16,70^ To date, the best antibiofilm peptides show broad-spectrum activity against biofilms formed by the most feared antibiotic resistant pathogens in our society and kill biofilms at concentrations of less than 1 μg ml^−1^. These peptides also work synergistically with antibiotics against multiple bacterial species^70^ and in model infections^71^ and are effective in animal and human organoid models of high density biofilm and abscess infections.^71^

Some of the key features identified to date are as follows. They have unusually broad spectra of activity, and the best peptides act against biofilm bacteria at concentrations substantially lower than those that inhibit planktonic bacteria.^4^ Critically, they can act either at the time of biofilm formation or when added after biofilms have matured for 1–2 days, and also have activity against mixed consortia of bacteria in biofilms, *e.g.* as observed for oral bacterial biofilms on hydroxyapatite substrates.^72^ Intriguingly in the latter studies, when added in combination with EDTA or chlorhexidine they can kill most oral bacteria in biofilms within 1–3 minutes.^73^ They act by targeting the alarmone ppGpp for degradation,^16,70^ but also trigger other mechanisms such as dispersal of biofilms,^74^ inhibition of attachment, *etc.* Such physical targets may make it difficult to obtain resistant mutants. Moreover, we submit that they might be very useful in dealing with biofilm heterogeneity. For example, stimulating dispersal might uncover the deeper layers of biofilms and decrease nutrient/oxygen deficiency at the base of the biofilm. Also, dispersal would revert biofilm bacteria back to the planktonic growth state and thus make them susceptible to conventional antibiotic therapy. Since a major target of these peptides is the stringent stress response that is known to regulate persister formation,^75^ it is also likely that with regards to biofilms, such peptides will inhibit the onset of this particularly insidious form of resistance. Lastly, HDPs as a class both stimulate protective immunity, as well as suppress inflammatory responses.^64^ This latter characteristic is particularly important since chronic inflammatory responses are a feature associated with chronic biofilm infections and are both pathological as well antagonizing effective host defences. For these reasons, antibiofilm peptides show potential as a fundamentally new approach against biofilm infections.

### The effects of antibiofilm peptides in *in vivo* biofilm infection models

As the study of biofilms and antibiofilm peptides expands, the requirement for cost-effective, simple and relevant *in vivo* models to test their efficacy is of the utmost importance. Biofilms are high density infections and it is already known that antibiotics are usually poorly effective when bacteria are present at very high local concentrations. Given the high costs to manufacture HDPs, their susceptibility to proteolytic cleavage, potential toxicity, and unknown interactions with body chemistry, the leap from *in vitro* experiments to *in vivo* assays is a large and critical step.^76–78^*In vitro* experiments and especially biofilm assays are critical in developing and identifying antibiofilm peptides and in our experience the establishment of standard and relatively predictive *in vitro* assays is critical.^79^ However, most *in vitro* assays are not performed under conditions that reflect the environment of mammalian biofilm infections and thus, have substantial limitations. For this reason, it is important that investigations rapidly move to predictive *in vivo* biofilm models. Many different *in vivo* biofilm models have been described to date although these tend to be complex, require considerable experimental manipulation as well as skills and, in particular, few have been used in conjunction with HDPs.^80,81^ As our understanding of biofilm biology and pathology has improved over time, so has the sophistication of *in vivo* models and future models will increasingly rely on extrapolation to actual biofilm infections. The 2019 Biofilm Bash^82^ concluded that it is important to select appropriate biofilm models that are relevant to *in situ* situations. For example, one must consider the pathogen, host, and disease, and use a model that is applicable to these parameters. It is not always easy to demonstrate the formation of biofilms *in vivo*, or to address optimal treatment modalities, pharmacokinetics, formulation and toxicity. Although addressing such aspects is well established in systemic infection models, they are not as easily addressed in biofilm models.

### Murine cutaneous abscess infection model

Abscesses are collections of pus (host fluids and cells) that build up within a tissue in the body. It must be emphasized that they are not *per se* biofilms but share the following features: (a) they are often associated with a high local density of bacteria; (b) deep tissue biofilms are embedded in biofilm-like matrices;^83^ (c) abscess pathology is directed by the stringent response;^84^ (d) they are confined to specific locations; (e) infections are associated with local inflammation^85^ and (f) they are extremely difficult to treat with antibiotics. Thus, the murine abscess model was established as a surrogate for a biofilm infection model due to these mechanistic similarities and ease of establishment and use. In this model, bacteria are injected subcutaneously into the back or hind flank of mice^85^ and peptides or antibiotics can be added intra-peritoneally or intra-abscess after infection.^85,86^ The antibiofilm peptide DJK-5, with only moderate antimicrobial properties, was able to reduce abscess size as well as bacterial recovery in an *S. aureus* USA300 (MRSA) and *P. aeruginosa* LESB58 model.^86^*In vitro* studies demonstrated that DJK-5 works by binding and sequestering the alarmone ppGpp thus inhibiting the stringent response which is required for biofilm formation^70^ as well as abscess formation.^84,86^ It was speculated that DJK-5 also targets other mechanisms besides the stringent response since mutants that were deficient for ppGpp, a known DJK-5 target, still demonstrated a decrease in CFU and abscess size when treated with the peptide. This could be due to an alternative target/mechanism or an anti-infective immunomodulatory function.^84^ Furthermore, DJK-5 and IDR-1018 used in combination with antibiotics were able to reduce the abscess size and/or CFUs in abscesses created by all ESKAPE pathogens (*Enterococcus faecium*, *S. aureus*, *Klebsiella pneumoniae*, *Acinetobacter baumannii*, *P. aeruginosa*, and *Enterobacter cloacae*) and *Escherichia coli*.^71^ It was demonstrated *in vitro* that peptides lead to increase in membrane permeability and target the stringent response.^71^

### *In vivo* respiratory models

Bacterial biofilms appear to be involved in various respiratory tract infections including various pulmonary infections, as well as upper respiratory tract infections such as chronic rhinosinusitis, adenoiditis, chronic and recurrent middle ear infection, and recurrent tonsilitis.^7,87,88^*P. aeruginosa* biofilms infections are the leading cause of morbidity and mortality in cystic fibrosis (CF) patients.^7,89^ Chronic rhinosinusitis leads to 4.1 million physician visits annually and is often treated surgically.^90,91^ In most of these cases, classical treatment with antibiotics is problematic and often insufficient, indicating the need for alternative therapies.

There are several studies that have examined the eradication/inhibition activities of HDPs on bacteria in murine lung models, as summarized previously.^64,92^ For example, mice were inoculated with *P. aeruginosa* intranasally, and intravenously challenged 1 hour later with the cyclic synthetic peptide ZY4 followed by treatment twice per day for 3 days.^93^ A significant reduction in bacterial load was found in the ZY4-treated mice, compared to untreated. These results were consistent with *in vitro* studies showing that ZY4 displayed dose-dependent inhibition and eradication against both *P. aeruginosa* and *A. baumannii* biofilms. However, it is worth stating that the authors did not demonstrate bacterial biofilms in the lungs.

In a sinusitis model,^94^*P. aeruginosa* were surgically inoculated into the sinus cavity of rabbits, and these presumably formed biofilms for 7 days. They were then treated by irrigation with 400× MIC of tobramycin (400 μg ml^−1^), as a positive control, or 0.1, 0.5, or 2.5 mg ml^−1^ of the LL-37-derived peptide OP-145. The rabbits and resulting biofilms were monitored *via* CFU counts from saline lavages on days 0, 1, 3, 5, 7, and 10. Both the tobramycin control and 2.5 mg ml^−1^ OP-145 largely eradicated bacteria, although at this concentration OP-145 had deleterious effects (increased inflammation and cilia shedding) on the health of the sinus cavity.

### *In vivo* catheter and device models

Indwelling catheters and other medical devices can become infected with biofilms by various bacteria and these can result in degradation of the device or implant, and/or seed systemic infections, given that such devices are mainly used in critically ill patients.^95^ One approach to keep devices free of biofilms is to coat their surfaces with antimicrobial substances. Antimicrobial and antibiofilm peptides work excellently *in vivo* and a large study initially demonstrated that activity of so-called tethered peptides was sequence-dependent, while studies of the tethering of biotinylated peptide Tet052 to an avidin-coated surface revealed a 75-fold reduction of activity when the peptide was tethered compared to the free peptide.^96^ Such a strategy prevents the deposit of organisms to enable biofilm formation and has been tested in animal models where it worked reasonably well to inhibit biofilm formation.^97,98^ The critical variability from study to study is the nature of the peptide and the tethering strategy utilized to enable a high local concentration of active peptide that is stable over time. One such effective strategy uses the polymer brush technology with branched chain molecules on the surface that provide many points of peptide attachment.^99^ The tethering strategy is additionally dependent on the nature of the substrate that can vary for different devices from silicon to polyurethane to titanium, *etc.* Another consideration for device coating is the fact that host and bacteria derived factors can in principle deposit on surfaces and mask the antibacterial agent and thus serve as a base on which biofilms can grow. For this reason, it is increasingly considered that anti-fouling surfaces (that do not readily bind organic matter) should be used in conjunction with tethered peptides. In this regard, the synthetic peptide E6 was successfully tethered to anti-fouling polyurethane catheters and displayed biofilm inhibitory activities *in vitro* and *in vivo* when implanted in the mouse bladder percutaneously and inoculated with luminescent strains of either *P. aeruginosa* or *S. aureus* the following day.^99^ After four days, the HDP coated catheters showed 96% decrease in luminescence compared to the uncoated catheters, and this was further increased to 99% reduction after seven days.^99^ The coated catheters had a 4-fold decrease in CFUs *versus* the uncoated catheters and reduced the bacterial burden in the urine by 3-fold.^99^

Another simpler *in vivo* catheter model involves inserting catheters subcutaneously on the backs of mice.^100^ Once implanted, MRSA can be injected in the vicinity of the sterile catheters and treated with HDPs, twice per day for three days, to demonstrate biofilm inhibition. The synthetic d-peptide CPF-C2 was shown both to inhibit and eradicate biofilm formation on subcutaneous urinary catheters.^100^ Membrane depolarization and propidium iodide uptake assays indicated that CPF-C2 affects the membrane integrity of MRSA.^100^

### Contact lens models

Contact lens usage is extremely common (45 million people in the US alone) but predisposes users to microbial keratitis and infectious corneal ulcers. Microbes, and particularly bacteria, can reside on these lenses often in multispecies biofilms and these can serve as reservoirs for the establishment of eye infections.^101^ When covalently attached to contact lenses, the synthetic peptide melimine (based on melittin and protamine) inhibited *P. aeruginosa* and *S. aureus* from binding in *in vitro* assays and disrupted the membranes of both.^102^ The coated lenses were shown to reduce the symptoms from contact lens induced acute red eye caused by *P. aeruginosa* in a guinea pig model.^102^ Melimine coated lenses were also shown to inhibit *P. aeruginosa* induced microbial keratitis in rabbits.^103^ Additionally, they showed a significant reduction in contact lens induced peripheral ulcers due to *S. aureus* in a rabbit model.^102^ Melimine coated lenses were tested in a human clinical trial, and showed few adverse effects while retaining antimicrobial properties, but did show an increase in fluorescein binding indicating a decrease in corneal integrity in patients.^104^

### Murine abraded skin infection models

Biofilms are a major obstacle that must be overcome in many instances of chronic infections. The human skin hosts many organisms, but problems arise when the tissue is damaged by physical or chemical means, or by underlying disease, and pathogens can then form biofilms.^105,106^ Many *in vivo* biofilm models establish and culture bacterial biofilms on or within wounds, tissues or organs. Conversely, a mouse ulcer wound model was developed that first established biofilms on membrane filters placed on agar nutrient plates.^107^ The biofilm bacteria were recovered and inoculated into 24 h old full-thickness excisions through the panniculus carnosus on the back of hairless diabetic mice. Subsequently, the biofilms were treated with the bee venom peptide, melittin (100 μM), or tobramycin (400 μM) in a 0.5% agarose Tris-acetate–EDTA hydrogel followed by 24 h incubation. Both treatments worked *in vitro*, but *in vivo* only a combination of both showed a significant ∼4-fold reduction in bacterial bioluminescence.^107^

The synthetic peptide, RP557 arose from three iterative design cycles based on LL-37, D2A21, and Tachyplesin-1.^108^ This peptide had strong *in vitro* activity against *P. aeruginosa* and *S. aureus* biofilms. Using a murine needle scratch skin wound model, affecting both the stratum corneum and epidermis, it was demonstrated that treatment with 0.2% RP557 in 2% hydroxypropyl methylcellulose was able to completely eliminate bioluminescent MRSA biofilms that formed within the wounds of control animals, and concurrently prevented mouse weight loss.

Another model is the so-called tape strip model that when performed with *S. aureus* clearly shows evidence of biofilm formation in histologically stained biopsies of murine dermal layers.^109^ The LL-37-derived peptide, 2% SAAP-148 was formulated in a 3.75% hypromellose gel base and applied to bacterially infected murine abraded skin.^110^ This SAAP-148 ointment was able to eradicate MRSA from 67% and 87% of mice in 24 and 48 h infection models. The same SAAP-148 treatment was also able to completely eradicate 24 and 48 h *A. baumannii* infections.

### Organoid and air–liquid interface models

The jump from *in vitro* to *in vivo* is substantial, relatively expensive, ethically challenging, and requires special training and facilities to conduct research. Recent developments in *ex vivo* organoid models are allowing researchers to move into models that enable screening in human tissue/organ surrogates instead of more complex animal models that do not accurately mirror our species dependent differences including innate and adaptive responses to antimicrobial agents.^111^ As a caveat, human organoid models also lack cellular complexity and immune responses as well as aspects of pharmacokinetics observed in animal models, but nevertheless are well suited for secondary screening studies. For example, the above-described study on SAAP-148 (ref. 110) also utilized an *ex vivo* human skin organoid model to demonstrate efficacy equivalent to that observed in the mouse abraded skin model.

The synthetic peptide WLBU-2 showed efficacy in a septicemia model and had biofilm inhibitory activity in crystal violet assays.^112,113^ Lashua *et al.* (2016) used a lung air–liquid interface (ALI) model derived from CF epithelial cells to demonstrate that WLBU-2 retained antibiofilm activity against *P. aeruginosa*. Biofilms were established on the ALI model for 1 h, unattached cells removed, and biofilms treated with increasing concentrations of WLBU-2 that inhibited *P. aeruginosa* in a dose-dependent manner with increased activity compared to LL-37. This peptide also exhibited efficacy against clinical *P. aeruginosa* isolates as well as biofilms formed on a CF ALI model.^113^

In another study, a lung ALI model was inoculated with *P. aeruginosa* at the apical site, unattached cells were removed after 1 h and 16 μM SPLUNC1-derived HDPs, α4 or α4-short, were added for 5 h.^114^ Following the treatment, enumeration of biofilm bacteria showed that α4-short could inhibit and eradicate biofilms. However, it must be stated that neither timing nor experimental conditions allowed a clear conclusion as to whether this model can truly be considered a biofilm model.^114^

## Conclusions

Given the lack of biofilm-selective therapies and the growing socio-economic burden of chronic infections, HDPs offer an exciting treatment approach for biofilm infections. Most studies indicating clinical efficacy to date have pursued topical treatments, which is especially promising in the context of skin and burn wound infections. The potent anti-inflammatory activity of HDPs combined with their broad-spectrum antibiofilm activity makes them promising drug candidates against a wide array of pathogenic biofilm infections. We submit that research aiming at improving *in vivo* and *ex vivo* biofilm infection models can drive the translation of HDPs alone or in combination with conventional antibiotics into clinical application.

## Conflicts of interest

The peptide research of REWH has been filed for patent protection by his employer, the University of British Columbia and licensed to ABT Innovations Inc, in which he has an ownership position.

## Supplementary Material
